# The TLR3/TICAM-1 signal constitutively controls spontaneous polyposis through suppression of c-Myc in *Apc*^*Min/+*^ mice

**DOI:** 10.1186/s12929-017-0387-z

**Published:** 2017-10-17

**Authors:** Junya Ono, Hiroaki Shime, Hiromi Takaki, Ken Takashima, Kenji Funami, Sumito Yoshida, Yohei Takeda, Misako Matsumoto, Masanori Kasahara, Tsukasa Seya

**Affiliations:** 1 Department of Vaccine Immunology, Hokkaido University Graduate School of Medicine, Kita 15, Nishi 7, Kita-ku, Sapporo, 060-8638 Japan; 2 Department of Microbiology Immunology, Hokkaido University Graduate School of Medicine, Sapporo, Japan; 3 Department of Pathology I, Hokkaido University Graduate School of Medicine, Sapporo, Japan

**Keywords:** TLR3, TICAM-1 (TRIF), c-Myc, Intestinal polyposis

## Abstract

**Background:**

Intestinal tumorigenesis is promoted by myeloid differentiation primary response gene 88 (MyD88) activation in response to the components of microbiota in *Apc* ^*Min/+*^ mice. Microbiota also contains double-stranded RNA (dsRNA), a ligand for TLR3, which activates the toll-like receptor adaptor molecule 1 (TICAM-1, also known as TRIF) pathway.

**Methods:**

We established *Apc* ^*Min/+*^ *Ticam1* ^*−/−*^ mice and their survival was compared to survival of *Apc* ^*Min/+*^ *Myd88* ^*−/−*^ and wild-type (WT) mice. The properties of polyps were investigated using immunofluorescence staining and RT-PCR analysis.

**Results:**

We demonstrate that TICAM-1 is essential for suppression of polyp formation in *Apc* ^*Min/+*^ mice. TICAM-1 knockout resulted in shorter survival of mice compared to WT mice or mice with knockout of MyD88 in the *Apc* ^*Min/+*^ background. Polyps were more frequently formed in the distal intestine of *Apc* ^*Min/+*^ *Ticam1* ^*−/−*^ mice than in *Apc* ^*Min/+*^ mice. Infiltration of immune cells such as CD11b^+^ and CD8α^+^ cells into the polyps was detected histologically. CD11b and CD8α mRNAs were increased in polyps of *Apc* ^*Min/+*^ *Ticam1* ^*−/−*^ mice compared to *Apc* ^*Min/+*^ mice. Gene expression of inducible nitric oxide synthase (iNOS), interferon (IFN)-γ, CXCL9 and IL-12p40 was increased in polyps of *Apc* ^*Min/+*^ *Ticam1* ^*−/−*^ mice. mRNA and protein expression of c-Myc, a critical transcription factor for inflammation-associated polyposis, were increased in polyps of *Apc* ^*Min/+*^ *Ticam1* ^*−/−*^ mice. A *Lactobacillus* strain producing dsRNA was detected in feces of *Apc* ^*Min/+*^ mice.

**Conclusion:**

These results imply that the TLR3/TICAM-1 pathway inhibits polyposis through suppression of c-Myc expression and supports long survival in *Apc* ^*Min/+*^ mice.

**Electronic supplementary material:**

The online version of this article (10.1186/s12929-017-0387-z) contains supplementary material, which is available to authorized users.

## Background

Tumor progression is closely linked to inflammation [1]. The intestine contains microorganisms that influence the incidence of inflammation-associated cancer via toll-like receptors (TLRs) expressed in gut mucosal cells. TLR2/4 and Nod-like receptor (NLR)1 are expressed in mucosa and detect intestinal bacterial patterns [2]. Nod1 signal may ameliorate inflammation-induced polyposis through nuclear factor (NF)-κB and activator protein 1 (AP1). Nod1 is reportedly important for maintaining the integrity of the intestinal epithelium to protect it against injury, inflammation and subsequent carcinogenesis [3]. TLRs except for TLR3 activate the adaptor MyD88, which serves as a key factor for promotion of carcinogenesis and development in colon cancer [4, 5]. TLR2/4 respond to bacteria and modulate NF-κB activation during the inflammatory response in the TLR/MyD88 pathways [5]. Other receptors which activate MyD88 also participate in inflammation and tumorigenesis in intestinal epithelial cells [6, 7], suggesting a crucial role for MyD88 in homeostasis of intestine. Epithelial cells and microflora work together cooperatively to maintain mucosal homeostasis via MyD88 signaling.

Several reports have suggested that lactobacillus produces partial or structural double-stranded (ds) RNA, which can activate TLR3 in the intestine [8]. TLR3 is expressed in mucosal epithelial cells and myeloid cells, which may sample the bacterial by-products of dsRNAs via phagocytosis [9]. TLR3 couples with the adaptor TICAM-1 (TRIF) to activate transcription factors, IRF3 and AP1. If this is the case, both MyD88 and TICAM-1 pathways participate in polyposis under the presence of complex innate stimulation. To test the relationship between TLR3/TICAM-1 and intestinal polyposis, we employed the *Apc* ^*Min/+*^ mouse model [10]. We found TICAM-1 is important for suppression of tumorigenesis and homeostasis of innate sensing of bacteria. We herein addressed the mechanism by which TLR3/TICAM-1 participates in polyp formation in *Apc* ^*Min/+*^ mice.

## Methods

### Mice

*Apc* ^*Min/+*^ mice on a C57BL/6 background were purchased from the Jackson Laboratory. *Myd88* ^*−/−*^ C57BL/6 mice were provided from Dr. S Akira (Osaka University). *Ticam1* ^*−/−*^ C57BL/6 mice were established in our laboratory. *Apc* ^*Min/+*^ mice were crossed to *Myd88* ^*−/−*^ or *Ticam1* ^*−/−*^ mice to generate *Apc* ^*Min+/−*^ *Myd88* ^*+/−*^ *, Apc* ^*Min+/−*^ *Myd88* ^*−/−*^ *, Apc* ^*Min+/−*^ *Ticam1* ^*+/−*^ and *Apc* ^*Min/+*^ *Ticam1* ^*−/−*^ littermates. Mice were bred and maintained under specific pathogen–free conditions. No abnormal behavior was observed in *Apc* ^*Min/+*^ *Ticam1* ^*−/−*^ during the period we maintained. In several individuals, growth retardation was observed for unknown reason, but we used individuals with normal body weight. Female and male mice were used for the present experiments. All animal experiments were approved by the University’s Committee on Use and Care of Animals.

### Harvesting of polyps

Mice were sacrificed by cervical dislocation. The small and large intestines were harvested and washed with cold PBS. The intestines were longitudinally slit open to grossly count tumors with the aid of a magnifier and stereomicroscope. Polyps ≧2 mm were collected by forceps and used for the following experiments.

### RT-qPCR and PCR

For quantitative PCR, total RNA was extracted with TRIzol, and 0.4 μg of RNA was treated with DNase I, and then reverse-transcribed using the High Capacity cDNA Transcription Kit (ABI) with random primers according to the manufacturer’s instructions. qPCR was performed using the Step One Real-Time PCR system (ABI). The RNA expression levels were normalized to *Gapdh*. To detect genomic DNA of *Lactobacillus Johnsonii*, DNA was extracted with QIAamp Stool Mini kit (Qiagen) form feces according to the manufacturer’s instructions. Purified genomic DNA was subjected to PCR using specific primers for *Lactobacillus Johnsonii*. PCR product was confirmed as amplified *Lactobacillus Johnsonii* genome by sequencing. The primers for detection of this bacillus were described in an early report [11]. Primer sequences used in this study are listed in Additional file 1: Table S1.

### Immunofluorescence

The small intestines were fixed with 4% paraformaldehyde (PFA)/PBS for 1 h at 4 °C. Fixed tissues were impregnated with 15% sucrose/PBS for 1 h following 30% sucrose/PBS for overnight at 4 °C with rotation. Tissues were then embedded in O.T.C. compound (Sakura Finetek Japan) and the frozen tissue blocks were sectioned by using cryotome (LEICA CM1850). Sections were fixed with acetone on ice for 30 min. After three washes with PBS, the sections were blocked with mouse serum IgG in 5% BSA/PBS for 1 h at R.T. Sections were stained with FITC-labeled anti-CD11c, anti-CD11b, anti-CD4 or anti-CD8, and mounted with Prolong Gold (Thermo Fisher Scientific). Samples were monitored at × 63 or 40 magnification using an LSM510 META microscopy (Zeiss).

### Flow cytometry

Single cell suspensions isolated from small intestine were stained with fluorescent dye-labeled Abs after blocking with an anti-CD16/32 Ab [12]. The following Abs were used: FITC- or APC/Cy7-CD45 (30-F11), PE-anti-CD11b (M1/70), APC-anti-CD11c (N418), APC-anti-CD3e (145-2C11), FITC-anti-CD4 (GK1.5), PE-anti-CD8a (53-6.7) (Biolegend). Dead cells were stained with 7AAD (Sigma). Samples were analyzed by a FACS Calibur or FACS Aria II (BD Bioscience); data analysis was performed using Flow Jo (Tree star).

### SDS-PAGE/western blotting

Proteins were extracted from polyps by SDS-containing sample buffer (125 mM Tris-HCl, pH 6.8, 4% SDS, 35% glycerol, and BPB). The samples were resolved on SDS-PAGE (7.5 or 10% gel), and blotted onto PVDF membranes (Millipore). Proteins were detected by rabbit antibodies against c-Myc and GAPDH (Cell signaling technology). The blot was labeled with Horseradish peroxidase-conjugated goat Ab against rabbit Ig’s (Biosource). The color was developed by ECL Prime Western Blotting Detection Reagent (GE Healthcare).

### Statistical analysis

*P*-values were calculated with Student t-test. Error bar represent the standard deviation (SD) between samples.

## Results

### Ticam1 deficiency results in short survival of *Apc*^*Min+*^ mice

**Fig. 1:**
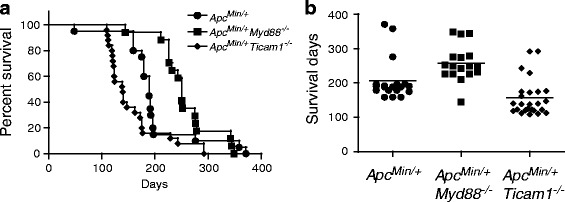
TICAM1 deficiency results in short survival of *Apc* ^*Min/+*^ mice. Kaplan-Meier survival curves of *Apc* ^*Min/+*^ (*n* = 20, circle), *Apc* ^*Min/+*^ *Myd88* ^*−/−*^ (*n* = 17, square), *Apc* ^*Min/+*^ *Ticam1* ^*−/−*^ (*n* = 25, diamond) mice. Panel **a**: survival curves of mice; Panel **b**: survival days of individulal mice

TLR transmits signal through two major adaptors, MyD88 and TICAM-1 [13]. We generated *Apc* ^*Min/+*^ mice on MyD88- or Ticam1-deficient background by crossing *Apc* ^*Min/+*^ mice to *Myd88* ^*−/−*^ or *Ticam1* ^*−/−*^ mice, respectively. The average survival of *Apc* ^*Min/+*^ mice was 28.6 weeks. *Apc* ^*Min/+*^ *Myd88* ^*−/−*^ mice survived longer than *Apc* ^*Min/+*^ mice, consistent with previous reports [7]. In contrast, survival times for *Apc* ^*Min/+*^ *Ticam1* ^*−/−*^ mice were significantly shorter at ~20 weeks (Fig. 1a, b). *Apc* ^*Min/+*^ *Myd88* ^*+/−*^ and *Apc* ^*Min/+*^ *Ticam* ^*+/−*^ mice showed similar life-spans, the average survival was 29 weeks indistinguishable from that of *Apc* ^*Min/+*^ mice (Additional file 2: Figure S1), suggesting the Ticam1 gene disruption was the event that affected life span. Only homologous deficiency of MyD88 or TICAM-1 affected the survival time.

**Fig. 2:**
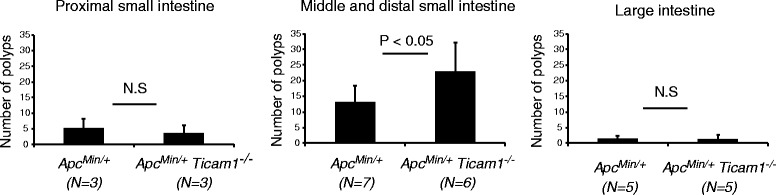
The number of polyps is significantly increased in *Apc* ^*Min/+*^ *Ticam1* ^*−/−*^ mice. The small and large intestines were collected from 23 to 29-weeks-old mice. The small intestine was divided to three equal parts, proximal small intestine, middle and distal small intestine, and large intestine. Polyps (≧ 2 mm) were counted in the indicated mice. The graphs show the number of polyps in proximal small intestine, distal small intestine, and large intestine. Error bars show SD. *p* < 0.05 in Student’s *t*-test and n.s.; not significant

Multiple polyposis occurs in the intestine of *Apc* ^*Min/+*^ mice, which represents intestinal tumorigenesis by adenomatous polyposis coli (APC) under the regulation of TLR signal [7]. *Apc* ^*Min/+*^ *Myd88* ^*−/−*^ mice reportedly have fewer polyps in the intestine than *Apc* ^*Min/+*^ mice [7]. We then examined the polyp formation in *Apc* ^*Min/+*^ *Ticam1* ^*−/−*^ mice at 23~29 weeks of age (Fig. 2). *Apc* ^*Min/+*^ mice showed a high frequency of polyp formation as reported previously [6, 7]. *Apc* ^*Min/+*^ *Ticam1* ^*−/−*^ mice had more polyps than *Apc* ^*Min/+*^ mice in the distal small intestine (Fig. 2). Thus, the incidence of tumor formation is high in the middle and distal intestine of *Apc* ^*Min/+*^ *Ticam1* ^*−/−*^ mice compared to *Apc* ^*Min/+*^ mice. Only a few polyps were observed in the proximal intestine and colon of *Apc* ^*Min/+*^ mice, and no or minimal increases in polyp numbers was observed in *Apc* ^*Min/+*^ *Ticam1* ^*−/−*^ mice (Fig. 2).

### Immune cell infiltration in polyps

**Fig. 3:**
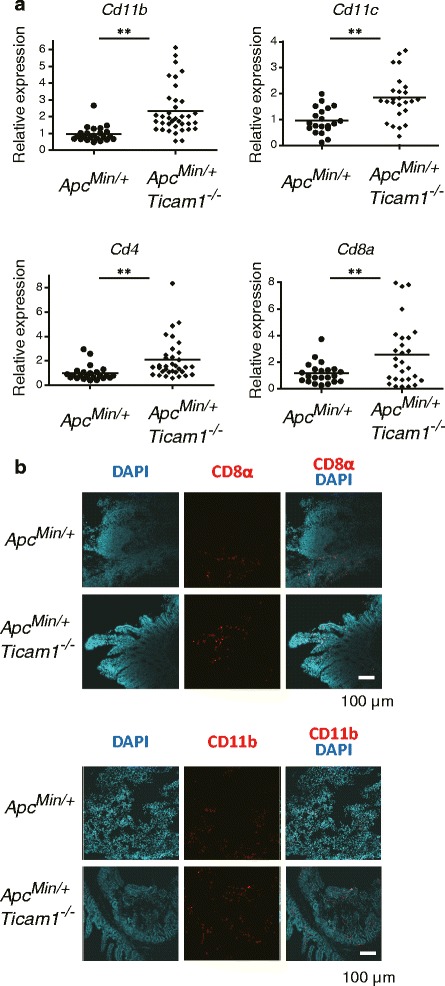
Immune cells infiltrate into polyps in *Apc* ^*Min/+*^ *Ticam1* ^*−*/−^ mice. **a** Gene expression in polyps prepared from 20 to 25-weeks-old mice were quantified by RT-qPCR. More than 3 mice in each group were used. **; *p* < 0.01 in Student’s t-test. **b** Immune staining of the small intestine prepared from 20 to 25-weeks-old mice using anti-CD11b and anti-CD8α antibodies. Data shows representative results of two independent experiments

TLRs on epithelial cells recognize microbial products of commensal bacteria and induce inflammatory responses, including oncogene expression [14]. TLRs except TLR3 provoke MyD88 signaling and accelerate the proliferation of intestinal epithelial cells and prohibit apoptosis [15]. We focused on immune cells infiltrating into mucosal polyps. CD8 and CD4 mRNAs were minimally detected in unaffected mucosa in *Apc* ^*Min/+*^ and were slightly increased in *Apc* ^*Min/+*^ *Ticam1* ^*−/−*^ mice at ~22 weeks age (Fig. 3a). CD11b and CD11c mRNAs levels were more increased in polyps of *Apc* ^*Min/+*^ *Ticam1* ^*−/−*^ mice than *Apc* ^*Min/+*^ mice (Fig. 3a). mRNA for CD4 and CD8α, markers of myeloid cells, were also increased in *Apc* ^*Min/+*^ *Ticam1* ^*−/−*^ polyps (Fig. 3a). We detected more accumulation of CD8α-positive cells in polyps of *Apc* ^*Min/+*^ *Ticam1* ^*−/−*^ mice compared to *Apc* ^*Min/+*^ mice by immunohistological staining (Fig. 3b). Similar tendencies were obtained with anti-CD11b antibody. The FACS profiles of the CD4-, CD8-, CD11b- and CD11c-positive cells in the intestine of *Apc* ^*Min/+*^ *Ticam1* ^*−/−*^ mice vs. *Apc* ^*Min/+*^ mice are shown in Additional file 3: Figure S2. The mRNAs of these immune cells were only marginally increased in the normal (non-polyp) region of the small intestine in *Apc* ^*Min/+*^ and *Apc* ^*Min/+*^ *Ticam1* ^*−/−*^ polyps (Additional file 4: Figure S3).

**Fig. 4:**
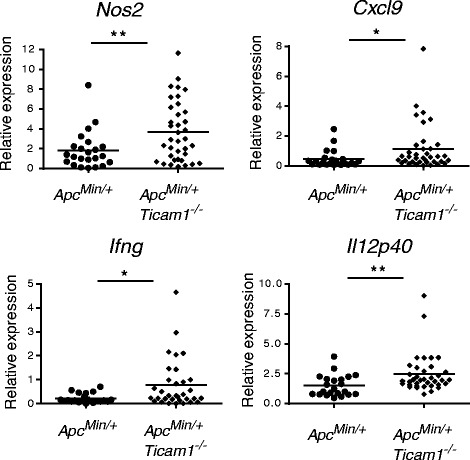
Inflammation is promoted in polyps of *Apc* ^*Min/+*^ *Ticam1* ^*−*/−^ mice. Gene expression in polyps prepared from 20 to 25-weeks-old mice were quantified by RT-qPCR. More than 3 mice in each group were used. *; *p* < 0.05, **; *p* < 0.01 in Student’s t-test

Inflammatory parameters were also increased in the polyps of *Apc* ^*Min/+*^ *Ticam1* ^*−/−*^ mice (Fig. 4). mRNA expression of iNOS (Nos2) was highly induced in *Apc* ^*Min/+*^ *Ticam1* ^*−/−*^ polyps compared to *Apc* ^*Min/+*^ polyps (Fig. 4). In addition, mRNA expression of CXCL9 (Cxcl9), IFN-γ (Ifng) and IL-12p40 (Il12p40) was slightly but significantly increased. In the normal (non-polyp) region, this tendency was not prominent (data not shown). Thus, tumor-related inflammation was induced to a greater extent in the intestine of *Apc* ^*Min/+*^ *Ticam1* ^*−/−*^ mice than in *Apc* ^*Min/+*^ mice.

### High expression of c-Myc in *Apc*^*Min/+*^*Ticam1*^*−/−*^ polyps

**Fig. 5:**
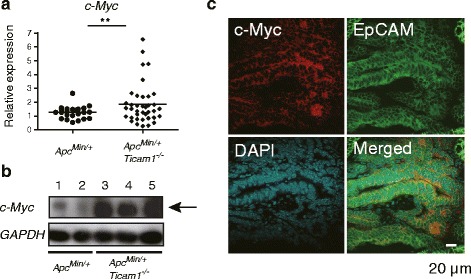
High expression of c-Myc in polyps of *Apc* ^*Min/+*^ *Ticam1* ^*−*/−^ mice. **a** Gene expression in polyps prepared from 20 to 25-weeks-old mice were quantified by RT-qPCR. More than 3 mice in each group were used. **; *p* < 0.01 in Student’s t-test. **b** Protein was extracted from pooled three polyps in distal small intestine and separated by SDS-PAGE. Expression levels of c-Myc protein were detected by Western blotting. GAPDH is used as an internal control. Each lane shows results from pooled samples prepared from individual mice. Lane 1-2: polyps from *Apc* ^*Min/+*^ mice, Lane 3-5: polyps from *Apc* ^*Min/+*^ *Ticam-1* ^*−/−*^ mice. **c** Immune staining of polyps in small intestine prepared from 20 to 25-weeks-old *Apc* ^*Min/+*^ *Ticam1* ^*−*/−^ mice using anti-c-Myc and anti-EpCAM antibodies. Data show representative results of two independent experiments

**Fig. 6:**
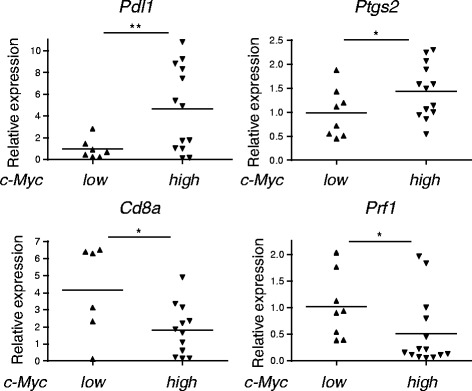
Differential gene expression in c-Myc^low^ and c-Myc^high^ polyps. Polyps prepared from 20 to 25-weeks-old *Apc* ^*Min/+*^ *Ticam1* ^*−*/−^ mice were divided into two groups according to the high (≧ 2.0) and low (<2.0) expression levels of *c-Myc* and gene expression was compared by RT-qPCR. *; *p* < 0.05, **; *p* < 0.01 in Student’s t-test

Previous reports suggest that c-Myc mRNA is not increased in epithelial cells in response to MyD88 activation [7, 16]. Instead, β-catenin signaling is amplified by constitutive inactivation of APC and transcriptionally up-regulates the c-Myc mRNA [17]. We next checked the levels of the c-Myc mRNA in *Apc* ^*Min/+*^ and *Apc* ^*Min/+*^ *Ticam1* ^*−/−*^ polyps. The levels of c-Myc mRNA were high in *Apc* ^*Min/+*^ *Ticam1* ^*−/−*^ polyps compared to *Apc* ^*Min/+*^ polyps (Fig. 5a). The c-Myc protein was abundant in *Apc* ^*Min/+*^ *Ticam1* ^*−/−*^ polyps in comparison with *Apc* ^*Min/+*^ polyps (Fig. 5b). The results were confirmed with confocal analysis (Fig. 5c). Although the staining density does not reflect the protein levels, the c-Myc protein level appears high in *Apc* ^*Min/+*^ *Ticam1* ^*−/−*^ polyps in Fig. 5c (and data not shown). The mRNA levels of PD-L1 (*Pdl1*) and COX2 (*Ptgs2*) appeared higher in c-Myc^high^ polyps than c-Myc^low^ polyps. Conversely, expression of CD8α (*Cd8a*) and Perforin-1 (*Prf1*) in c-Myc^high^ polyps was lower than c-Myc^low^ polyps. c-Myc^high^ polyps are likely to form a microenvironement favorable for tumor growth (Fig. 6). The TLR3 level was barely affected by environment in c-Myc^low^ and c-Myc^high^ polyps (not shown): the genes affected by environment in c-Myc^low^ vs. c-Myc^high^ polyps are shown in Additional file 5: Table S2.

### Intestine of *Apc*^*Min/+*^ mouse contains bacteria with TLR3-stimulating capacity

**Fig. 7:**
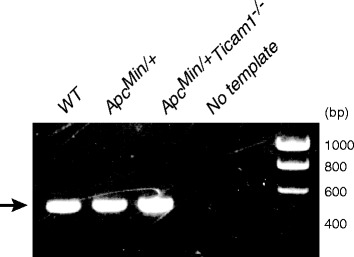
Detection of genomic DNA of *Lactobacillus Johnsonii* in feces. Genomic DNA of *Lactobacillus Johnsonii* in feces was detected by PCR using specific primers for *Lactobacillus Johnsonii* genome. Data shows representative results of two independent experiments

Since TICAM-1 is the adaptor of TLR3 and TLR4 [5, 13], c-Myc may be suppressed by TLR3/4-TICAM-1 signaling. A previous report suggested that TLR3 is activated in response to dsRNA moieties yielded by *Lactobacillus* in mouse intestine [8]. PCR analysis using the specific primer sets detected the genome DNA of *Lactobacillus johnsonii* in the feces of *Apc* ^*Min/+*^ mice, implying that the TLR3 signaling is constitutively activated in the intestine (Fig. 7).

## Discussion

Carcinogenesis is established through multi-step gene mutations in intestinal epithelial cells. Loss-of-function of APC occurs in most patients of familial-associated polyposis [18] and causes malignant polyposis. *Apc* ^*Min/+*^ mice have a mutation in the APC gene and accelerate polyposis in the intestine but not colon [10]. While *Apc* ^*Min/+*^ mice die ~24 weeks from complication of tumorigenesis, their survival is prolonged by MyD88 disruption [7]. Thus, MyD88 signal of TLRs enhances protumor activity to shorten the survival. TICAM-1 transmits the other signal to activate a transcription factor IRF3. Here we showed that knockout of TICAM-1 results in short survival in *Apc* ^*Min/+*^ mice, which suggests that the TLR3/TICAM-1 signal reduces polyposis promoted by the TLR2/4/MyD88 signaling pathway.

Intestinal epithelial cells express TLRs which utilize two adaptors, MyD88 and/or TICAM-1, as well as immune cells and tumor cells in mice [5, 13]. MyD88 evokes inflammatory signal that causes nuclear translocation of NF-κB and regulates apoptosis in tumor cells. Liberation of inflammatory cytokines sustains tumor-supporting microenvironment. In the *Apc* ^*Min/+*^ mouse model of intestinal tumorigenesis, activation of the MyD88 pathway is related to stabilization of c-Myc protein but not to up-regulation of its mRNA in epithelial cells, resulting in a decrease in tumor growth in *Apc* ^*Min/+*^ *Myd88* ^*−/−*^ mice [7, 16]. On the other hand, the role of TICAM-1 in the regulation of c-Myc expression and tumorigenesis has been controversial [19, 20]. TICAM-1 has been identified in myeloid cells including dendritic cells and several subsets of macrophages [21–23]. The TLR3/TICAM-1 pathway takes part in cross-priming and IL-12 production that in turn causes DC priming and cytotoxic T cell (CTL) induction [22, 24, 25]. Moreover, some tumor cell lines express TLR3 [26]. We show that c-Myc expression is suppressed via TICAM-1: TICAM-1 signals constitutively suppress c-Myc expression and TICAM-1 loss results in c-Myc up-regulation. Over-expression of c-Myc abrogates its regulatory function in the cell cycle and induces tumorigenesis [17]. TLR3 signaling suppresses tumor cell growth through down-regulation of c-Myc [19]. The c-Myc level, however, barely affect the TLR3 expression. Thus, it would be reasonable to hypothesize that the TICAM-1 signaling pathway suppress c-Myc transcription and reduces intestinal polyp formation in *Apc* ^*Min/+*^ mice. We have examined Ticam1-associated gene clusters by comprehensive method [27]. So, we selected inflammatory-induced genes form the Ticam1-associated genes (Additional file 5: Table S2). PolyI:C-activated TLR3-TICAM-1 signaling also suppresses tumor growth via immune activation [28]. Hence, TLR3 ligand may bimodally act on TLR3 expressed in tumor cells and immune cells, leading to tumor regression.

Our results imply that constitutive activation of the TLR3/TICAM-1 signaling pathway occurs in intestinal mucosa of *Apc* ^*Min/+*^ mice. TLR3 stimulation also occurs with *Lactobacillus* dsRNA in the intestine [8]. In this scenario, bacterial-derived dsRNA behaves like a tumor suppressor via c-Myc regulation through the TLR3 signaling pathway. Thus, *Lactobacillus* may support good flora conditions to constitutively activate TLR3 in the intestinal epithelial cells or immune cells. TLR3/TICAM-1 signaling is likely to suppress c-Myc mRNA expression through direct stimulation of TLR3 on epithelial cells or by indirect stimulation via TLR3-expressing immune cells. Further study is required to elucidate the mechanism of TICAM-1-mediated suppression of c-Myc expression through intestinal microflora.

Many reports suggest that MyD88 induces protumor signal in tumor or transformed cells, but in dendritic cells MyD88 induces priming of T cells to regress tumor cells (28). Myeloid-derived suppressor cells and tumor-associated macrophages express TLR2 that activates MyD88 signaling to promote invasion and metastasis [29]. However, TLR3/TICAM-1 signals convert these myeloid cells to tumoricidal effectors in tumor microenvironment [30, 31]. Even in epithelial and tumor cells, stimulation of TLR3 does not promote cell growth or inflammation, which may be attributable to suppression of c-Myc. TLR3 adjuvant is now considered more successful in tumor immunotherapy compared to other TLR adjuvants. This study demonstrates an additional advantage of TLR3 adjuvant for direct therapeutic application to tumor cells: TLR3-targeted therapy may be of benefit to cancer patients by acting on both immune cells and tumor microenvironment.

## Conclusion

The TLR3/TICAM-1 signaling suppresses c-Myc mRNA expression through direct stimulation of TLR3 in intestinal cells to suppress mucosal polyposis in *Apc* ^*Min/+*^ mice. Survival time is shortened by knockout of Ticam-1 in *Apc* ^*Min/+*^ mice.

## Additional files

Additional file 1: Table S1. Primer sequences used for real-time RT-PCR. (DOCX 19 kb)

Additional file 2: Figure S1. Survival curve and days of ApcMin/+ mice. Survival curves (upper panel) and survival days (lower panel) were monitored in ApcMin/+, ApcMin/+Myd88−/+ and ApcMin/+Ticam1−/+ mice. *N* > 18 in each group. (PDF 2353 kb)

Additional file 3: Figure S2. FACS analysis of immune cells in the small intestine. We checked the degrees of infiltration of immune cells into small intestine in ApcMin/+Ticam1−/− mice by FACS analysis. The whole small intestine was harvested from WT, ApcMin/+ and ApcMin/+ Ticam1−/− mice. The proportions of small intestine-infiltrating CD11b+, CD11c+, CD4+ T and CD8+ T cells were evaluated on FlowJo ver.9.9.4 (Tree Star). (PDF 456 kb)

Additional file 4: Figure S3. Immune cell markers in the non-polyp region of the distal intestine. Gene expression in the non-polyp region of the distal intestine prepared from 20 to 25-weeks-old ApcMin/+ mice (*n* = 3) or ApcMin/+Ticam1−/− mice (*n* = 5) was quantified by RT-qPCR. n.s.; not significant in Student’s t-test. (PDF 68 kb)

Additional file 5: Table S2. Relative expression levels of inflammatory-associated genes in c-Myclow and c-Mychigh polyps. (DOCX 19 kb)

## Abbreviations

AP1: Activation protein 1
APC: Adenomatous polyposis coli
CTL: Cytotoxic T lymphocytes
IFN: Interferon
IL: Interleikin
IRF: Interferon regulatory factor
MyD88: Myeloid differentiation primary response gene 88
Nod1: Nucleotide-binding oligomerization domain-containing protein 1
TICAM-1: TIR domain-containing adapter molecule 1
TLR: Toll-like receptor
