# Supplementary material for: The TLR3/TICAM-1 signal constitutively controls spontaneous polyposis through suppression of c-Myc in ApcMin/+ mice
Source: J Biomed Sci. 2017 Oct 17;24:79. doi: 10.1186/s12929-017-0387-z (PMC5646017; doi:10.1186/s12929-017-0387-z)
Supplement: Supplementary file 1 — Primer sequences used for real-time RT-PCR. (DOCX 19 kb) [file 12929_2017_387_MOESM1_ESM.docx]

**Table S1.** Primer sequences used for real-time RT-PCR.

| **Gene** | **Primer sequences** | |
| --- | --- | --- |
|  | **Forward** | **Reverse** |
| *Gapdh* | 5’-GCCTGGAGAAACCTGCCA-3’ | 5’-CCCTCAGATGCCTGCTTCA-3’ |
| *Cd4* | 5’-TCCTTCCCACTCAACTTTGC-3’ | 5’-AAGCGAGACCTGGGGTATCT-3’ |
| *Cd8a* | 5’-GCTCAGTCATCAGCAACTCG-3’ | 5’-ATCACAGGCGAAGTCCAATC-3’ |
| *Cd11b* | 5’-GCAGTCATCTTGAGGAACCGTGTC-3’ | 5’-GTTGGTATTGCCATCAGCGTCC-3’ |
| *Cd11c* | 5’-ATGTTGGAGGAAGCAAATGG-3’ | 5’-CCTGGGAATCCTATTGCAGA-3’ |
| *c-Myc* | 5’-ATGCCCCTCAACGTGAACTTC-3’ | 5’-GTCGCAGATGAAATAGGGCTG-3’ |
| *Cxcl9* | 5’-GATAAGGAATGCACGATGCTC-3’ | 5’-TCTCCGTTCTTCAGTGTAGCAA-3’ |
| *Ifng* | 5’-GATATCTGGAGGAACTGGCAAAAG-3’ | 5’-AGAGATAATCTGGCTCTGCAGGAT-3’ |
| *Il12p40* | 5’-AATGTCTGCGTGCAAGCTCA-3’ | 5’-ATGCCCACTTGCTGCATGA-3’ |
| *Nos2* | 5’-CAGCTGGGCTGTACAAACCTTC-3’ | 5’-CATTGGAAGTGAAGCGTTTCG-3’ |
| *Pdl1* | 5’-GCTGAAAGTCAATGCCCCATA-3’ | 5’-TCCACGGAAATTCTCTGGTTG-3’ |
| *Prf1* | 5’-CAAGGTAGCCAATTTTGCAGC-3’ | 5’-GGCGAAAACTGTACATGCGAC-3’ |
| *Ptgs2* | 5’-AGACAACATAAACTGCGCCTTTT-3’ | 5’-GGATGTGAGGAGGGTAGATCATCT-3’ |
| *L.johnsonii* | 5'-TCGAGCGAGCTTGCCTAGATGA-3’ | 5'- TCCGGACAACGCTTGCCACC-3’ |
