# Supplementary figures and images for: The TLR3/TICAM-1 signal constitutively controls spontaneous polyposis through suppression of c-Myc in ApcMin/+ mice
Source: J Biomed Sci. 2017 Oct 17;24:79. doi: 10.1186/s12929-017-0387-z (PMC5646017; doi:10.1186/s12929-017-0387-z)

Fig.S1

A

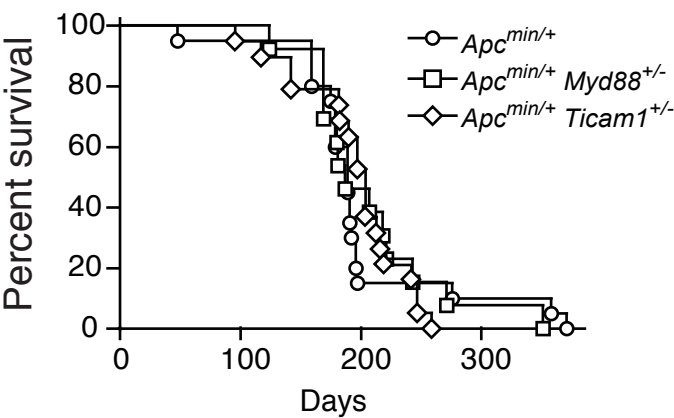

B

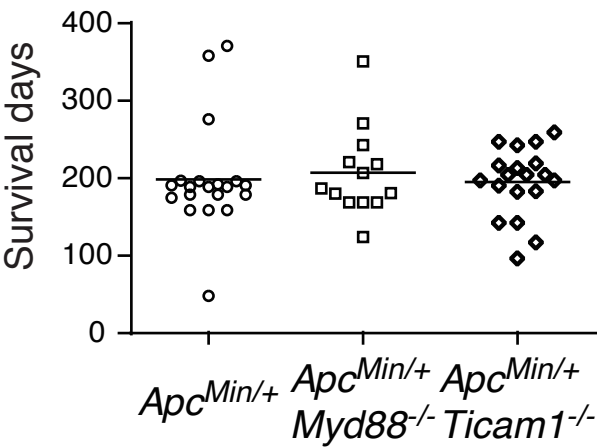

Supplement: Supplementary file 2 — Survival curve and days of ApcMin/+ mice. Survival curves (upper panel) and survival days (lower panel) were monitored in ApcMin/+, ApcMin/+Myd88−/+ and ApcMin/+Ticam1−/+ mice. N > 18 in each group. (PDF 2353 kb) [file 12929_2017_387_MOESM2_ESM.pdf]

Fig.S2      Small intestine-infiltrating immune cells

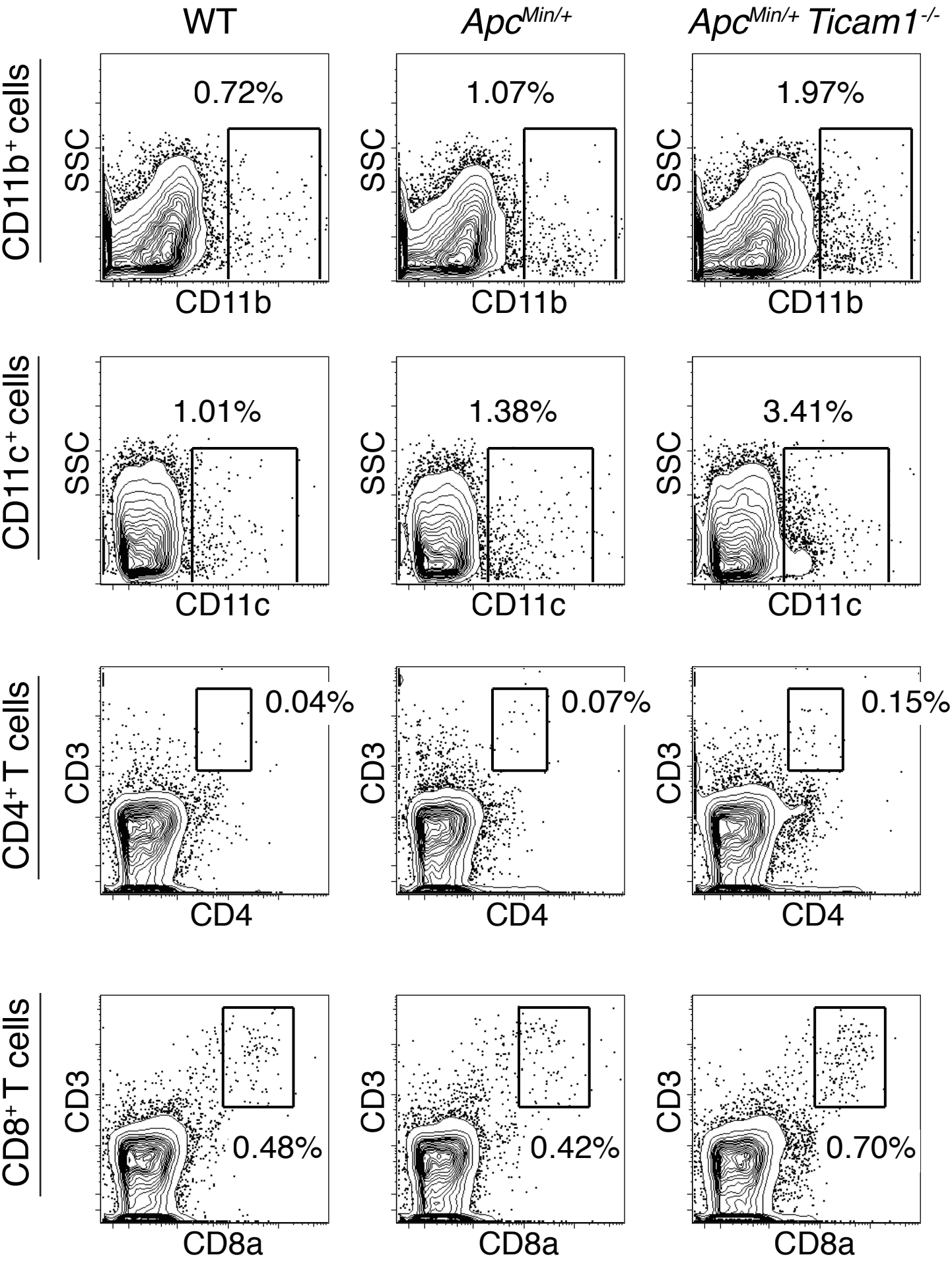

Supplement: Supplementary file 3 — FACS analysis of immune cells in the small intestine. We checked the degrees of infiltration of immune cells into small intestine in ApcMin/+Ticam1−/− mice by FACS analysis. The whole small intestine was harvested from WT, ApcMin/+ and ApcMin/+ Ticam1−/− mice. The proportions of small intestine-infiltrating CD11b+, CD11c+, CD4+ T and CD8+ T cells were evaluated on FlowJo ver.9.9.4 (Tree Star). (PDF 456 kb) [file 12929_2017_387_MOESM3_ESM.pdf]

Fig.S3

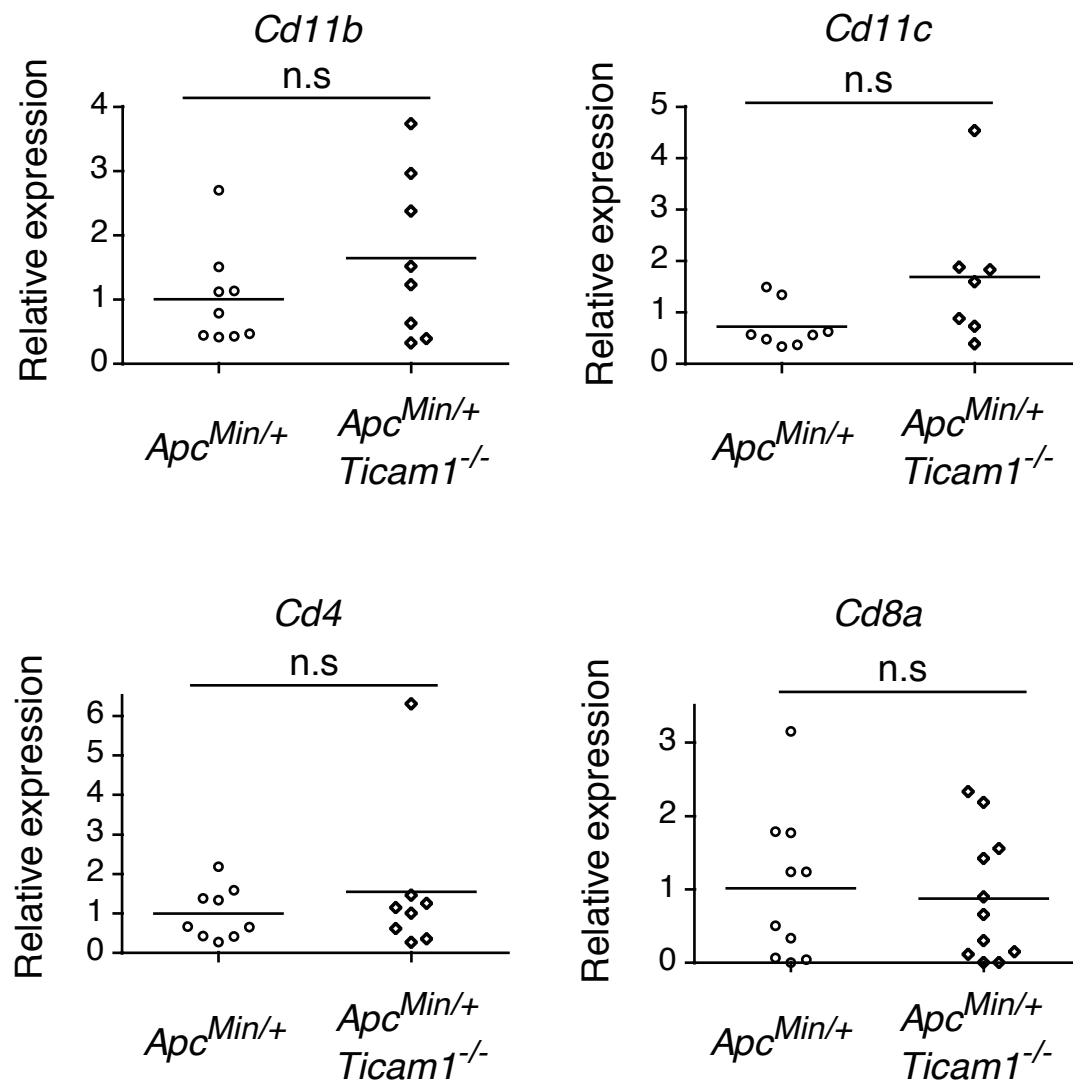

Supplement: Supplementary file 4 — Immune cell markers in the non-polyp region of the distal intestine. Gene expression in the non-polyp region of the distal intestine prepared from 20 to 25-weeks-old ApcMin/+ mice (n = 3) or ApcMin/+Ticam1−/− mice (n = 5) was quantified by RT-qPCR. n.s.; not significant in Student’s t-test. (PDF 68 kb) [file 12929_2017_387_MOESM4_ESM.pdf]
