# Supplementary material for: The TLR3/TICAM-1 signal constitutively controls spontaneous polyposis through suppression of c-Myc in ApcMin/+ mice
Source: J Biomed Sci. 2017 Oct 17;24:79. doi: 10.1186/s12929-017-0387-z (PMC5646017; doi:10.1186/s12929-017-0387-z)
Supplement: Supplementary file 5 — Relative expression levels of inflammatory-associated genes in c-Myclow and c-Mychigh polyps. (DOCX 19 kb) [file 12929_2017_387_MOESM5_ESM.docx]

**Table S2.** Relative expression levels of inflammatory-associated genes in c-Myc^low^ and c-Myc^high^ polyps

| **Gene** | ***Apc^Min/+^ Ticam1^-/-^*** |
| --- | --- |
|  | **Fold induction of gene expression**  **(c-Myc^high^ / c-Myc^low^)** |
| *Cd4* | 0.70 |
| *Cd8a* | 0.54***** |
| *Cd11b* | 1.31 |
| *Cd11c* | 0.81 |
| *Nos2* | 1.91 |
| *Arg1* | 9.15***** |
| *Il17a* | 2.34***** |
| *Il12p40* | 1.37 |
| *Ifng* | 0.61 |
| *Ptgs2* | 1.46***** |
| *Tnfa* | 3.16***** |
| *Il6* | 2.00 |
| *Gzmb* | 0.35***** |
| *Prf1* | 0.50***** |
| *Mmp9* | 0.44***** |

* ; *p*<0.05 (c-Myc^high^ vs. c-Myc^low^ ) in Student’s t-test
